# Seed-Mediated Gene Flow Promotes Genetic Diversity of Weedy Rice within Populations: Implications for Weed Management

**DOI:** 10.1371/journal.pone.0112778

**Published:** 2014-12-01

**Authors:** Zhuoxian He, Xiaoqi Jiang, Disna Ratnasekera, Fabrizio Grassi, Udugahapattuwage Perera, Bao-Rong Lu

**Affiliations:** 1 Ministry of Education Key Laboratory for Biodiversity and Ecological Engineering, Department of Ecology and Evolutionary Biology, Fudan University, Shanghai, China; 2 Department of Agricultural Biology, Faculty of Agriculture, University of Ruhuna, Matara, Sri Lanka; 3 Botanical Garden, Department of Biology, University of Bari, Bari, Italy; 4 Department of Bioscience, University of Milan, Milano, Italy; East China Normal University, China

## Abstract

Increased infestation of weedy rice—a noxious agricultural pest has caused significant reduction of grain yield of cultivated rice (*Oryza sativa*) worldwide. Knowledge on genetic diversity and structure of weedy rice populations will facilitate the design of effective methods to control this weed by tracing its origins and dispersal patterns in a given region. To generate such knowledge, we studied genetic diversity and structure of 21 weedy rice populations from Sri Lanka based on 23 selected microsatellite (SSR) loci. Results indicated an exceptionally high level of within-population genetic diversity (*H_e_* = 0.62) and limited among-population differentiation (*F_st_* = 0.17) for this predominantly self-pollinating weed. UPGMA analysis showed a loose genetic affinity of the weedy rice populations in relation to their geographical locations, and no obvious genetic structure among populations across the country. This phenomenon was associated with the considerable amount of gene flow between populations. Limited admixture from STRUCTURE analyses suggested a very low level of hybridization (pollen-mediated gene flow) between populations. The abundant within-population genetic diversity coupled with limited population genetic structure and differentiation is likely caused by the considerable seed-mediated gene flow of weedy rice along with the long-distance exchange of farmer-saved rice seeds between weedy-rice contaminated regions in Sri Lanka. In addition to other effective weed management strategies, promoting the application of certified rice seeds with no weedy rice contamination should be the immediate action to significantly reduce the proliferation and infestation of this weed in rice ecosystems in countries with similar rice farming styles as in Sri Lanka.

## Introduction

Weeds continue to pose great challenges for crop productivity around the world, despite of decades of intensive efforts and practices for weed control and eradication [1],[2]. One of the major characteristics of weeds attributed to their success is the tremendous diversity represented by various weedy populations, with respect to their morphology, reproduction, insect and pathogen responses, and underlying genetic variation [3]. Genetic diversity, together with phenotypic plasticity, allows weeds to exploit novel and diverse opportunities as they occur and infest in agro-ecosystems [1]. Therefore, designing relevant strategies is necessary for the effective control and management of different types of weeds in agro-ecosystems. This is particularly true for weeds that are conspecific to crops, meaning that weeds and crops are the same biological species. The control and management of conspecific weeds become extremely complicated and difficult because these weeds can easily acquire genetic variation from crops through natural hybridization and introgression, through which genes from a crop variety can be transferred to a weedy population [4]. Such a process can facilitate the accumulation of genetic diversity and adaptive evolution of conspecific weeds in human disturbed agro-ecosystems. It is thus necessary to generate knowledge concerning the genetic diversity, divergence, and their spatial distribution of a particular weedy species and its populations. In addition, it is important to reveal the underlying mechanisms that influence the spread and accumulation of such genetic diversity, either through pollination or seed dispersal, in a given geographical region.

Weedy rice (*Oryza sativa* f. *spontanea*: Poaceae) is a noxious weed infesting rice fields worldwide. This weed is the same biological species of cultivated rice (*O. sativa*), with annual and predominantly inbreeding characteristics [5],[6]. Weedy rice can fiercely compete with the cultivated rice occurring in the same field for nutrients and other resources [7],[8], causing considerable yield losses and grain-quality reduction of the crop rice [5],[9]. Once infested in rice fields, it is difficult to remove and control weedy rice, because of its strong seed shattering and seed dormancy, which allows weedy rice to persist in soil seed-banks for a long period of time [5],[10]. Similar to its cultivated counterpart, weedy rice is an predominantly inbreeding taxon with a low outcrossing rate (<2%) [5],[6],[11]. However, this mating system enable weedy rice to mimic phenotypic and physiologic traits of cultivated rice through natural hybridization with and allelic introgression from its co-existing rice cultivars under selection [5],[6]. Such introgression can also enhance genetic diversity of weedy rice populations that have acquired genes from different rice varieties at various times. The crop-to-weed gene introgression makes it difficult to distinguish weedy rice plants from cultivated rice plants in the same fields [11]–[13]. It also makes the control of weedy rice using a uniform measure very difficult [5]. In addition, weedy rice can produce a large number of seeds at maturity through self-pollination, and some weedy rice seeds can be accidentally mixed in cultivated rice seeds during harvesting, which may promote the long-distance dispersal of weedy rice seeds by farmers, along with the exchange of the cultivar seeds between regions [5]. Therefore, it is particularly important to develop unique weed management strategies to minimize the impact of weedy rice on rice production based on the increased understanding of its genetic diversity and distribution [14].

As the most important staple crop in Sri Lanka, rice provides ∼45% of total calorie requirement and ∼40% of total protein requirement for Sri Lankan people [15]. A large number of traditional and improved rice varieties are grown in nearly all agro-ecological systems of the country, except for the agricultural regions with elevations higher than 2000 m [16]. This fact evidently reflects the significance of sufficient rice production as food security in Sri Lanka. However, the infestation of weedy rice has become a great threat to the sustainable production of cultivated rice since the report of this weed in 1980s when different weedy rice populations were found in eastern and northern parts of Sri Lanka [17],[18]. The surveys made by the Sri Lankan Department of Agriculture in the Ampara District in the late 1990s showed that weedy rice seriously infested ∼200 ha of cultivated rice fields, accounting for ∼0.3% of total rice cultivation land in this District [18]. However, weedy rice are currently expanded to nearly all rice planting regions across the country [9],[19]. The estimated rice yield losses caused by the infestation of weedy rice varied largely from 40%∼90%, depending on its density in rice fields [18]. Based on the fact that Sri Lanka has yet to achieve self-sufficiency in rice production, the quick expansion of weedy rice become inevitable threats in most rice-growing areas in this country. Effective weedy rice control must be taken into immediate consideration for securing rice production.

Previous studies of morphological traits demonstrated significant within- and among-population diversity in Sri Lankan weedy rice [9]. The morphological variation patterns suggested a possibility of seed-mediated gene flow between different rice planting regions through farmers' frequent exchanges of cultivated rice seeds—one of the reasons that promoted significant weedy rice infestation in the country [9]. Usually, farmers from a particular region grow the same rice variety for 10∼15 year and they save their own rice seeds for the next cultivation season. There is a common practice that farmers exchange rice seeds between villages and regions throughout the country, which suggests the possibilities of long-distance dispersal of mixed weedy rice seeds along with the movement of cultivated rice seeds. Such a rice seed management is commonly practiced in many other developing countries, including China, India, and Indonesia. Hypothetically, such long-distance seed dispersal may result in the following genetic effects: (1) promoting genetic diversity of a weedy rice population (in the same rice field) by acquiring different genotypes from various sources/regions, and (2) disturbing the formation of the isolation-by-distance pattern of weedy rice populations across a spatial distance [20], which may alter the genetic structure and relationships of the weedy rice populations. However, such a hypothesis of long-distance seed dispersal of weedy rice promoted by farmers' exchange of rice cultivars needs supports from population genetic studies applying molecular markers.

To estimate genetic diversity and structure of weedy rice populations, a set of 23 selected microsatellite (SSR) markers were used to study weedy rice populations collected from a broad range of rice cultivation regions across Sri Lanka. The primary objective of this study is to address the following questions: (1) Does abundant within-population genetic diversity exist in Sri Lankan weedy rice? (2) Is there any spatial genetic structure or differentiation among weedy rice populations across the country? (3) Does long-distance gene flow occur among weedy rice populations, which is associated with genetic diversity of Sri Lankan weedy rice? The answer of these questions will provide a useful guideline for designing effective strategies to control and manage weedy rice in Sri Lanka and in other countries that have similar or the same rice farming styles and seed management as in Sri Lanka.

## Materials and Methods

### Plant materials

**Figure 1:**
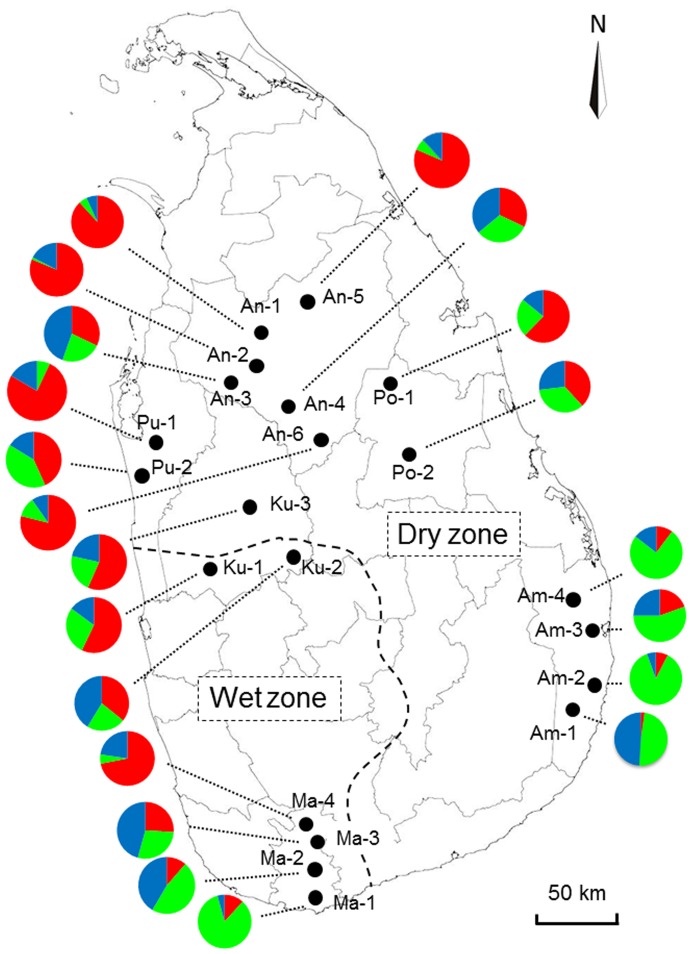
Geographical locations of the 21 weedy rice populations collected from Sri Lanka. The population codes at each collection site are shown in Table 1. Division of the Dry and Wet zones [9] is represented by the dotted line. The colors in pies are corresponded to those in the STRUCTURE analysis for weedy rice populations.

Twenty-one weedy rice (*Oryza sativa* f. *spontanea*) populations with a total of 449 individuals sampled in the “Yala” (May∼August) season from the Dry and Wet zones of Sri Lankan major rice planting regions in 2009 were included in this study (Table 1, Figure 1). Given that weedy rice is a type of weeds occurring in rice fields, no special permission is required to collect these samples. For each population, mature panicles from 13 to 30 weedy rice individuals with a spatial interval of ∼10 m were collected. This strategy aimed to avoid sampling of the same genotypes from a weedy rice population in the same rice field, because the natural seed dispersal of wild rice per season was only a few meter. Information about population codes, the number of sampled individuals, locations, ecological zones, and GPS readings of these weedy rice populations is indicated in Table 1. In addition, nine rice (*O. sativa*) varieties (each with three plants) and 15 wild rice (*O. nivara*) accessions (each with one plant) provided by the National Rice Research and Development Institute of Sri Lanka were used as references in analyses. The rice varieties were At308, At362, Bg300, Bg352, Bg358, Bg379-2, Bg379-25, Suduheenati, and Suwandal. All weedy rice seed samples were deposited in the Department of Agricultural Biology, Faculty of Agriculture, University of Ruhuna, Sri Lanka.

**Table 1:** The twenty-one weedy rice populations collected from different geographical locations in Sri Lanka with their GPS readings.

Population Code	No. of individuals	Locality (District/Village)	Ecological Zone	Location (GPS)	
				Latitude (N)	Longitude (E)
Am-1	30	Ampara/Lahugala	Dry	06° 52′ 08.30″	81° 43′ 01.85″
Am-2	17	Ampara/Pothuvil	Dry	06° 55′ 09.45″	81° 49′ 27.53″
Am-3	24	Ampara/Akkaraipattu	Dry	07° 12′ 34.80″	81° 47′ 11.50″
Am-4	27	Ampara/Malwatta	Dry	07° 19′ 17.30″	81° 43′ 56.19″
Po-1	19	Polonnaruwa/Hingurakgoda	Dry	08° 02′ 13.30″	80° 57′ 03.40″
Po-2	27	Polonnaruwa/Nawanagaraya	Dry	07° 54′ 43.40″	81° 00′ 44.50″
An-1	30	Anuradhapura/Saliyapura	Dry	08° 23′ 48.70″	80° 26′ 39.80″
An-2	17	Anuradhapura/Thalawa	Dry	08° 12′ 10.70″	80° 20′ 17.40″
An-3	13	Anuradhapura/Thambuttegama	Dry	08° 07′ 51.89″	80° 17′ 34.27″
An-4	15	Anuradhapura/Maha-Iluppallama	Dry	08° 06′ 49.55″	80° 28′ 55.24″
An-5	30	Anuradhapura/Madawachchiya	Dry	08° 33′ 27.50″	80° 28′ 55.90″
An-6	21	Anuradhapura/Kekirawa	Dry	08° 02′ 05.66″	80° 36′ 29.70″
Ku-1	19	Kurunegala/Kuliyapitiya	Wet	07° 27′ 42.90″	80° 04′ 48.40″
Ku-2	18	Kurunegala/Mawathagama	Wet	07° 26′ 43.10″	80° 25′ 47.50″
Ku-3	22	Kurunegala/Bingiriya	Dry	07° 39′ 49.50″	80° 11′ 37.20″
Ma-1	27	Matara/Akurugoda	Wet	06° 02′ 39.30″	80° 33′ 48.30″
Ma-2	20	Matara/Mulatiyana	Wet	06° 09′ 21.00″	80° 34′ 30.50″
Ma-3	14	Matara/Pasgoda	Wet	06° 14′ 36.82″	80° 36′ 35.91″
Ma-4	22	Matara/Kotapola	Wet	06° 15′ 42.00″	80° 36′ 16.80″
Pu-1	18	Puttalam/Puttalam	Dry	08° 02′ 34.80″	79° 54′ 55.10″
Pu-2	19	Puttalam/Madampe	Dry	07° 32′ 03.40″	79° 46′ 44.50″

### DNA extraction, amplification and electrophoresis

DNA extraction was conducted at Fudan University, Shanghai of China, using the duplicates of the rice seed samples collected from Sri Lanka. Filled seeds from each individual were germinated in an illuminated incubator (27°C) with a light/dark cycle of 16/8 h in May, 2012. The total genomic DNA was extracted from fresh leaf tissues of a single plant at about 10-day seedling stage following a modified cetyltrimethyl ammonium bromide (CTAB) protocol [21].

Twenty three microsatellite (SSR) loci (Table 2) were selected for the analysis of genetic diversity, structure, and relationships of all included materials. The detailed information of the SSR loci is available at the GRAMENE website (http://archive.gramene.org/markers/). The forward primers of the 23 SSR primer pairs were fluorescently labeled with three different colors (FAM for blue, JOE for green, ROX for red; Invitrogen Inc., Shanghai, China) for the fluorescence detection of amplified DNA products (see Jiang et al. [6] for details).

**Table 2:** The 23 SSR primer pairs used in this study with detail information on their DNA sequences, motifs, molecular weight, and expected heterozygosity in the examined weedy rice materials.

Primer ID	*Ch* location	Forward Primer	Reverse Primer	SSR motif	Molecular weight (bp)	*H_e_*
RM220	1	GGAAGGTAACTGTTTCCAAC	GAAATGCTTCCCACATGTCT	(CT)_17_	100–122	0.39
RM263	2	CCCAGGCTAGCTCATGAACC	GCTACGTTTGAGCTACCACG	(CT)_34_	156–200	0.63
RM208	2	TCTGCAAGCCTTGTCTGATG	TAAGTCGATCATTGTGTGGACC	(CT)_17_	162–182	0.65
RM55	3	CCGTCGCCGTAGTAGAGAAG	TCCCGGTTATTTTAAGGCG	(GA)_17_	215–237	0.55
RM218	3	TGGTCAAACCAAGGTCCTTC	GACATACATTCTACCCCCGG	(TC)_24_ACT_5_(GT)_11_	118–152	0.35
RM241	4	GAGCCAAATAAGATCGCTGA	TGCAAGCAGCAGATTTAGTG	(CT)_31_	106–164	0.65
RM280	4	ACACGATCCACTTTGCGC	TGTGTCTTGAGCAGCCAGG	(GA)_16_	146–176	0.39
RM289	5	TTCCATGGCACACAAGCC	CTGTGCACGAACTTCCAAAG	G_11_(GA)_16_	86–150	0.60
RM249	5	GGCGTAAAGGTTTTGCATGT	ATGATGCCATGAAGGTCAGC	(AG)_5_A_2_(AG)_14_	102–144	0.44
RM276	6	CTCAACGTTGACACCTCGTG	TCCTCCATCGAGCAGTATCA	(AG)_8_A_3_(GA)_33_	100–148	0.40
RM253	6	TCCTTCAAGAGTGCAAAACC	GCATTGTCATGTCGAAGCC	(GA)_25_	115–145	0.64
RM234	7	ACAGTATCCAAGGCCCTGG	CACGTGAGACAAAGACGGAG	(CT)_25_	136–164	0.25
RM11	7	TCTCCTCTTCCCCCGATC	ATAGCGGGCGAGGCTTAG	(GA)_17_	112–142	0.66
RM149	8	GCTGACCAACGAACCTAGGCCG	GTTGGAAGCCTTTCCTCGTAACACG	(AT)_10_	228–270	0.71
RM44	8	ACGGGCAATCCGAACAACC	TCGGGAAAACCTACCCTACC	(GA)_16_	93–113	0.24
RM219	9	CGTCGGATGATGTAAAGCCT	CATATCGGCATTCGCCTG	(CT)_17_	192–232	0.62
RM215	9	CAAAATGGAGCAGCAAGAGC	TGAGCACCTCCTTCTCTGTAG	(CT)_16_	138–152	0.12
RM228	10	CTGGCCATTAGTCCTTGG	GCTTGCGGCTCTGCTTAC	(GA)_6_(GA)_36_	82–172	0.64
RM258	10	TGCTGTATGTAGCTCGCACC	TGGCCTTTAAAGCTGTCGC	(GA)_21_(GGA)_3_	122–152	0.65
RM21	11	ACAGTATTCCGTAGGCACGG	GCTCCATGAGGGTGGTAGAG	(GA)_18_	130–178	0.69
RM202	11	CAGATTGGAGATGAAGTCCTCC	CCAGCAAGCATGTCAATGTA	(CT)_30_	160–212	0.55
RM19	12	CAAAAACAGAGCAGATGAC	CTCAAGATGGACGCCAAGA	(ATC)_10_	214–254	0.67
RM235	12	AGAAGCTAGGGCTAACGAAC	TCACCTGGTCAGCCTCTTTC	(CT)_24_	100–138	0.47

*Ch*: Chromosome; *H_e_*: Nei's unbiased expected heterozygosity [23].

The polymerase chain reactions (PCR) were carried out on a 2720 Thermal Cycler (Applied Biosystems Inc., Foster, USA) with a 10 µl volume containing 1×buffer (with Mg^2+^), 0.2 mM each of dNTPs (TaKaRa Dalian, China), 0.2 µM each of SSR primers, 50 ng of genomic DNA template, and 0.4 U of Taq polymerase (Biocolor Biotech Inc., Shanghai, China). The reaction procedure started with denaturation at 94°C for 4 min, followed by 28∼30 cycles of 30 s at 94°C, 30 s at 55°C, and 40 s at 72°C, and ended with 7 min extension at 72°C.

According to the size and fluorophore color of the DNA fragments, the amplified PCR products from 4∼5 SSR primer pairs were added to the mixture of 9 µL Hi-Di Formamide (to denature the dsDNA) and internal lane size standard (GeneScan^TM^ -500 LIZ) for denaturing at 94°C for 5 min and then cooled at 4°C. The mixture was separated on ABI 3130xl capillary electrophoresis genotyper (Applied Biosystems Inc., Foster, USA).

### Data score and analysis

#### Data score for different SSR loci

The amplified DNA fragments were scored as genotype data for each (weedy, cultivated, or wild) rice sample, given the co-dominant nature of SSR markers. The amplified products were coded either as homozygous genotypes having two equally sized fragments or heterozygous genotypes having two different sized fragments, based on their molecular weight (bp) of the internal lane size standard (GeneScan-500LIZ). All the allelic scores were performed using the software GeneMarker ver. 2.4.2 (SoftGenetics LLC, State College, USA).

#### Estimating genetic diversity and differentiation of weedy rice populations

Genetic diversity was estimated for the 21 weedy rice populations with 449 individuals based on the 23 SSR loci. The calculated genetic parameters included: (1) number of observed allele per locus (*N_a_*); (2) number of effective alleles per locus (*N_e_*); (3) observed heterozygosity (*H_o_*); (4) Nei's unbiased expected heterozygosity (*H_e_*) [22]; and (5) fixation index (*F*). In addition, the analysis of molecular variance (AMOVA) was carried out to dissect the distribution of genetic diversity within and among weedy rice populations, and between the Dry and Wet zones [23]. All the above analyses were conducted using the software GenAlEx ver. 6.5 [24]. Genetic differentiation among all weedy rice populations included in this study was estimated as *F_st_* by FSTAT software (http://www2.unil.ch/popgen/softwares/fstat.htm) and subsequently transformed in *F*′*_st_*, a standardized measure of genetic differentiation [25]. The software RECODEDATA [26] was used to recode the data set so that each population had unique alleles, and thus estimating the maximum values of genetic differentiation, *F_st_* (max). The standardized values of genetic differentiation (*F′_st_*), were subsequently obtained by dividing each *F_st_* by the correspondent *F_st_* (max).

#### Determining genetic relationships of weedy rice populations

Genetic relationships of the 21 weedy rice populations were estimated based on genetic similarity generated from the original SSR data matrix. As references, rice varieties and wild rice accessions were included as two independent groups in the analysis. Unweighted pair-group method with arithmetic averages (UPGMA) was used to determine genetic divergence and relationships between weedy populations, in comparison with wild and cultivated rice groups, based on their similarity matrix. The cluster analysis was conducted using the software NTSYS ver. 2.02 [27].

To examine whether there existed an isolation-by-distance pattern, the Mantel test was conducted to examine the correlation between genetic distance of weedy rice populations and spatial distance of their geographical localities. The analysis was performed using the software GenAlEx ver. 6.5 [24].

#### Estimating gene flow and genetic structure of weedy populations

To estimate the level of gene flow, the pair-wise migrants per generation (*N_m_*) [28] among 22 populations, including 21 weedy rice populations and one cultivated rice group (9 varieties) were calculated. This estimation was under the assumption that two populations of size *N* drawn from a large pool of populations exchange a fraction *m* of migrants each generation, and that the mutation rate *u* is negligible as compared to the migration rate *m* [29],[30]. The gene-flow analysis was performed by calculating the *M* value using the software Arlequin ver. 3.5 [30]. To estimate the relationship between the level of genetic diversity and gene flow of a particular weedy rice population, correlation between the expected heterozygosity (*H_e_*) of the weedy rice population and the average number of migrants of the weedy rice population with all other paired populations was analyzed using the linear regression model installed in the SPSS software ver. 19.0.0 (http://www.spss.com/).

To analyze the genetic structure and possible admixture of weedy and cultivated rice, the Bayesian clustering algorithm based program STRUCTURE ver. 2.3.4 was used to assign the samples within a hypothetical K number of populations proposed by Pritchard et al. [31]. The genotypic data matrix of 22 populations generated from 23 SSR primer pairs was analyzed with 500 000 Markov chain Monte Carlo iterations after a burn-in of 10000 iterations under the admixture model and correlated allele frequencies [32]. The data matrix was run 8 times for each value of K ancestral populations, with K varying from 1 to 8. The appropriate K was inferred using the *ad hoc* statistic ΔK [33] (available on the website of http://taylor0.biology.ucla.edu/structureHarvester/). In addition, genetic structure of weedy rice populations was superimposed to their geographical locations using the frequencies of STRUCTURE file by Microsoft Excel (Figure 1).

## Results

### Genetic diversity and differentiation of weedy rice populations

The 21 weedy rice populations collected from Sri Lanka showed a considerably high level of overall genetic diversity (*H_e_* = 0.62) and within-population genetic diversity (*H_e_* = 0.37-0.69), although great diversity was detected among different populations (Table 3). Unexpectedly, a much greater proportion of genetic diversity was detected within populations than among populations across the whole country (Table 4). While very low genetic differentiation (*F_st_* 0.17) was detected among weedy rice populations (Table 4). In addition, low proportion of genetic differentiation (∼2%) was also detected between the Dry and Wet zones (Table 4).

**Table 3:** Parameters of genetic diversity in 21 weedy rice populations from Sri Lanka based on 23 SSR primer pairs.

Population code	*N_a_*	*N_e_*	*H_o_*	*H_e_*	*F*
Am-1	5.2 (0.2)	3.3 (0.3)	0.28 (0.03)	0.67 (0.04)	0.57 (0.04)
Am-2	3.2 (0.3)	2.8 (0.3)	0.15 (0.04)	0.61 (0.05)	0.78 (0.05)
Am-3	4.4 (0.4)	3.0 (0.4)	0.12 (0.03)	0.58 (0.06)	0.81 (0.04)
Am-4	4.5 (0.3)	2.8 (0.2)	0.12 (0.03)	0.61 (0.04)	0.82 (0.05)
Po-1	3.7 (0.3)	2.9 (0.3)	0.06 (0.02)	0.64 (0.05)	0.91 (0.03)
Po-2	4.5 (0.4)	3.4 (0.4)	0.09 (0.03)	0.66 (0.05)	0.87 (0.04)
An-1	3.2 (0.2)	2.0 (0.2)	0.04 (0.02)	0.43 (0.05)	0.93 (0.03)
An-2	3.3 (0.2)	2.2 (0.1)	0.04 (0.02)	0.54 (0.04)	0.90 (0.06)
An-3	3.1 (0.3)	2.6 (0.3)	0.06 (0.02)	0.58 (0.06)	0.81 (0.07)
An-4	4.0 (0.4)	3.6 (0.4)	0.07 (0.03)	0.70 (0.06)	0.88 (0.05)
An-5	4.8 (0.2)	2.4 (0.2)	0.10 (0.03)	0.52 (0.04)	0.82 (0.05)
An-6	4.0 (0.3)	2.7 (0.3)	0.07 (0.03)	0.58 (0.05)	0.89 (0.04)
Ku-1	4.0 (0.3)	2.9 (0.3)	0.14 (0.03)	0.58 (0.06)	0.78 (0.04)
Ku-2	3.4 (0.3)	2.7 (0.3)	0.06 (0.02)	0.58 (0.07)	0.90 (0.03)
Ku-3	4.7 (0.2)	3.7 (0.3)	0.07 (0.02)	0.74 (0.04)	0.89 (0.03)
Ma-1	3.5 (0.3)	2.4 (0.2)	0.08 (0.02)	0.52 (0.05)	0.88 (0.03)
Ma-2	3.3 (0.3)	2.6 (0.3)	0.06 (0.02)	0.56 (0.06)	0.89 (0.04)
Ma-3	3.2 (0.3)	2.7 (0.3)	0.05 (0.02)	0.57 (0.07)	0.89 (0.04)
Ma-4	4.2 (0.3)	2.4 (0.2)	0.01 (0.00)	0.55 (0.05)	0.98 (0.01)
Pu-1	3.7 (0.2)	2.7 (0.3)	0.04 (0.02)	0.59 (0.05)	0.93 (0.04)
Pu-2	4.3 (0.2)	3.4 (0.3)	0.11 (0.03)	0.72 (0.04)	0.84 (0.04)
Overall	11.8 (0.6)	3.7 (0.4)	0.10 (0.01)	0.64 (0.05)	0.85 (0.02)

*N_a_*: number of observed alleles; *N_e_*: number of effective alleles; *H_o_*: observed heterozygosity; *H_e_*: Nei's unbiased expected heterozygosity [23]; *F*: fixation index.

Numbers in parentheses indicate standard error.

**Table 4:** Analysis of molecular variance (AMOVA) of 21 weedy rice populations based on 23 SSR primer pairs.

Source	Df	SS	Var. comp.	%
Among Zones	1	88.45	0.14	1.8%
Among Populations	19	712.73	0.71	8.7%
Within Populations	877	6390.52	7.29	89.5%
Total	897	7191.70	8.14	100%

Df: degree of freedom; SS: sum of squared deviations; Var. comp.: variance component estimates; %: percentage of total variation.

Results indicated a total number of 272 alleles from the 23 selected SSR markers, all of which were polymorphic in the examined weedy rice samples, with an average of ∼11.8 alleles (*N_a_*) per locus. However, the number of effective alleles (*N_e_*) was much lower than the number of observed alleles (*N_a_*), with an average of 3.4 alleles per locus. This suggested a large number of rare alleles in Sri Lankan weedy rice samples. Obviously, each of the analyzed weedy rice populations presented a high level of genetic diversity as measured by *H_e_*, although varied considerably among weedy rice populations (Table 3). The highest genetic diversity was observed in a northern weedy rice population (An-4, *H_e_* = 0.69), even though a relatively less number of samples (15 individuals) were included for analysis. The lowest genetic diversity was also detected in a northern population (An-1, *H_e_* = 0.37), although 30 individuals were included to represent the population. There was no considerable difference in genetic diversity between populations from Dry zone and Wet zone (Table 1, 3). Except for population Am-1 (possibly with a wild rice origin), all weedy rice populations had extremely low level (0.01–0.06) of observed heterozygosity (*H_o_*) that was in good correlation the high level of fixation index (0.87∼0.99), indicating the self-pollination mating system and an extremely low hybridization rate of weedy rice. However, all the above results suggested the significantly high level of genetic diversity within weedy rice populations from Sri Lanka.

Noticeably, AMOVA analysis of the 21 weedy rice populations further indicated about 83% of genetic diversity that was distributed within the populations and 15% of genetic diversity among populations. Only 2% of genetic diversity was distributed among the Dry and Wet zones (Table 4), indicating nearly no genetic differentiation between the two zones. This pattern indicated an unexpectedly high level of within-population genetic diversity and generally a low level of among-population genetic differentiation as estimated by *F_st_* in Sri Lankan weedy rice (Table S1–2).

### Genetic relationship between weedy rice populations

**Figure 2:**
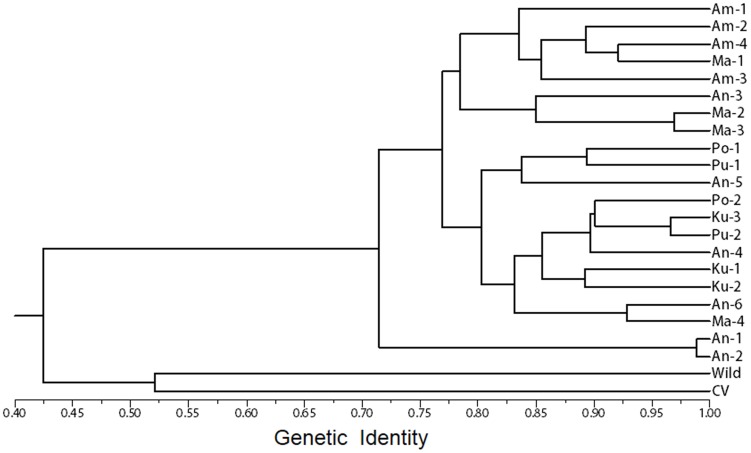
UPGMA dendrogram constructed based on the genetic similarity of 21 weedy rice populations. Similarity was estimated by 23 SSR loci, using cultivated (CV) and wild rice (Wild) as two reference groups.

To illustrate genetic relationships of the 21 Sri Lankan weedy rice populations across the country, a UPGMA dendrogram (Figure 2) was constructed based on the cluster analysis of the genetic similarity matrix generated from SSR genotyping of these populations. The two groups of nine rice varieties and 15 wild rice (*O. nivara*) accessions were included in the cluster analysis as references. The results of cluster analysis showed a clear two-group pattern, in which all the weedy rice populations included in the analysis were clustered as one group. This group was evidently separated from the cultivated- and wild-rice groups at the similarity coefficient value of 0.42. However, all the Sri Lankan weedy rice populations showed a relatively close but variable genetic relationship at the similarly coefficient values of ∼0.72–0.98. The cultivated and wild rice sub-groups were somehow distantly related, showing separation at the similarity coefficient value of 0.52.

Noticeably, the clustering of weedy rice populations did not follow the rule of “isolating-by-distance”. In other words, many weedy rice populations from the same geographical locations were not grouped together. This trend could be evidently reflected by some populations that were genetically distinct from other populations with a close spatial distance collected from the same geographical regions (Figure 1, 2). In contrast, some populations from different geographical locations showed a close genetic relationship and were grouped together (Figure 1, 2, Table 1). These results indicated the possible long-distance and frequent gene flow among the weedy rice populations from different geographical locations. The Mantel test did not show significant correlation (*r^2^* = 0.011, P<0.01) between the genetic-distance and spatial-distance matrices of the weedy rice populations, supporting the finding that there was neither obvious genetic structure nor pattern of isolation-by-distance (IBD) among the weedy rice populations across the country.

### Gene flow and genetic structure of weedy rice populations

**Figure 3:**
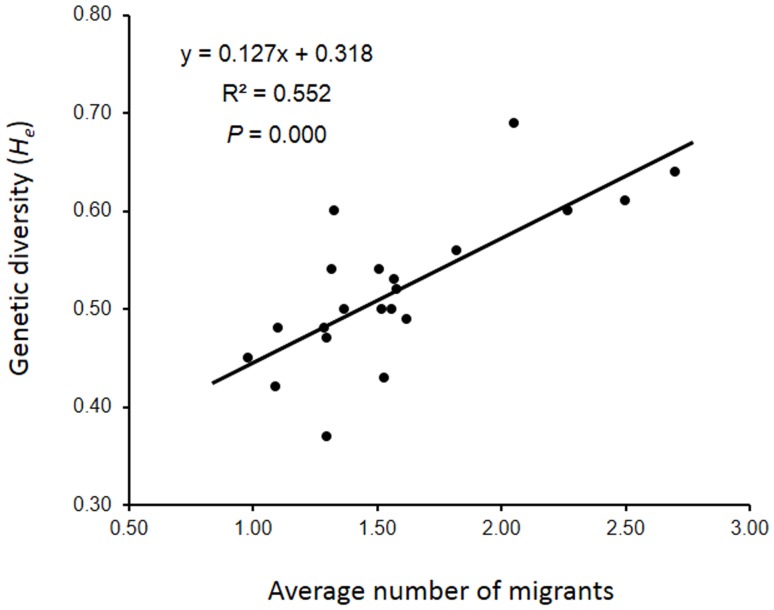
Correlation between genetic diversity and the average number of immigrants of a particular weedy rice population. Nei's (1987) unbiased expected heterozygosity (*H_e_*) [22] was used to estimate the correlation.

To estimate the level of possible gene flow between weedy rice populations, the number of migrants per generation (*N_m_*) between each pair of all populations was calculated using cultivated rice as a reference. Pair-wise migration data (Table 5) showed a considerably high level of gene flow among different weedy rice populations, with an average number of 1.27 migrants (*N_m_*) per generation, although significant variation was detected with a range between0.14-122. In addition, gene flow between weedy rice populations and cultivated rice was also detected with an average number of migrants per generation to be 0.17, ranging between 0.08-0.34. Correlation analysis indicated a significant positive correlation (*R^2^* = 0.552, P<0.001) between the expected heterozygosity (*H_e_*) and the average number of migrants per generation of a particular weedy rice population with other populations (Figure 3). This result suggested increased genetic diversity in a weedy rice population by gene flow.

**Table 5:** Number of pair-wise migrants per generation (*Nm*) between 21 weedy rice populations and cultivated rice group (CV) calculated based on the formula proposed by Slatkin and Barton [28].

	Am-1	Am-2	Am-3	Am-4	Po-1	Po-2	An-1	An-2	An-3	An-4	An-5	An-6	Ku-1	Ku-2	Ku-3	Ma-1	Ma-2	Ma-3	Ma-4	Pu-1	Pu-2	CV
Am-1	-																					
Am-2	0.72	-																				
Am-3	0.72	1.35	-																			
Am-4	1.00	1.85	1.19	-																		
Po-1	0.46	0.62	0.64	0.55	-																	
Po-2	0.70	1.63	1.43	1.47	1.04	-																
An-1	0.14	0.25	0.23	0.19	0.33	0.33	-															
An-2	0.19	0.29	0.33	0.25	0.48	0.49	-	-														
An-3	0.44	0.41	0.43	0.51	0.96	0.88	0.27	0.46	-													
An-4	0.61	1.28	0.80	0.58	1.40	2.04	0.38	0.52	0.71	-												
An-5	0.31	0.30	0.44	0.31	1.04	0.53	0.23	0.30	0.31	0.40	-											
An-6	0.28	0.42	0.55	0.45	0.77	1.46	0.34	0.45	0.29	0.55	0.57	-										
Ku-1	0.36	0.45	0.42	0.50	1.44	0.99	0.30	0.40	0.54	0.87	0.53	0.82	-									
Ku-2	0.58	0.67	0.50	0.53	0.76	1.21	0.21	0.26	0.49	1.64	0.44	0.49	1.50	-								
Ku-3	0.49	0.88	0.63	0.76	1.66	1.84	0.78	1.21	1.36	3.54	0.49	0.80	1.58	0.93	-							
Ma-1	0.77	1.08	0.51	1.83	0.55	0.82	0.20	0.23	0.50	0.52	0.30	0.37	0.60	0.61	0.61	-						
Ma-2	0.54	0.65	0.42	0.66	0.60	0.74	0.17	0.24	0.74	0.58	0.29	0.31	0.61	0.85	0.63	0.95	-					
Ma-3	0.56	0.64	0.46	0.65	0.72	0.87	0.24	0.33	1.43	0.72	0.31	0.32	0.85	1.13	0.90	0.87	122.76	-				
Ma-4	0.27	0.29	0.45	0.38	0.61	1.07	0.23	0.34	0.37	0.43	0.37	1.94	0.77	0.39	0.68	0.30	0.28	0.32	-			
Pu-1	0.26	0.29	0.28	0.31	1.53	0.39	0.25	0.34	0.38	0.55	0.50	0.34	0.77	0.39	1.14	0.27	0.29	0.29	0.28	-		
Pu-2	0.49	1.11	0.73	0.86	1.72	2.48	0.45	0.65	0.84	2.67	0.47	0.90	1.34	0.67	-	0.56	0.47	0.53	1.09	0.88	-	
CV	0.31	0.20	0.17	0.18	0.15	0.22	0.07	0.08	0.14	0.31	0.11	0.12	0.16	0.34	0.17	0.16	0.18	0.17	0.11	0.11	0.18	-

**Figure 4:**
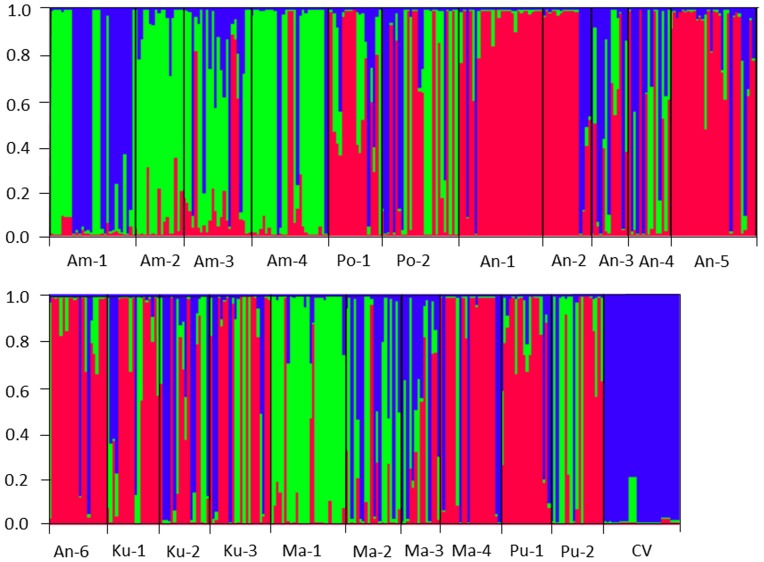
Genetic relationships of 21 weedy rice populations (Am-1 to Pu-2) and rice varieties (CV) from STRUCTURE analysis. Each individual is represented by a vertical bar assigned into three colors, with height proportional to each of the three inferred genetic components. The numbers beside vertical axis represent probability of assignment.

The STRUCTURE analysis generally indicated a relatively distinct genetic structure of weedy rice populations from the cultivated rice that was used as a reference, although the major component (indicated as the blue color assignment) of cultivated rice was also found in most of the weedy rice populations (Figure 4). The genetic structure was constructed on the basis of the analysis with the appropriate common ancestry (K) value determined to be 3 from the likelihood scores. Weedy rice populations were represented by three major genetic components (indicated as the red, green, and blue color assignments in Figure 4), with a certain degree of admixture of the three components for a few individuals that exhibited a multiple-cluster assignment. Weedy rice populations from Ampara (Am-1∼Am-4) and Matara (Ma-1∼Ma-3) Districts shared a similar genetic component, whereas populations from Polonnaruwa (Po-1, Po-2), Anuradhapura (An-1∼An-6), Kurunegala (Ku-1∼Ku-3), Matara (Ma-4), and Puttalam (Pu-1, Pu-2) Districts shared another similar genetic component. Noticeably, some weedy rice populations, such as Am-1, An-2, An-3, Ma-2, and, Ma-3 shared a relatively high proportion of genetic cluster assignment with the cultivated rice (Figure 4), probably indicating the frequent gene flow from cultivated rice to these weedy rice populations or their origin from cultivated rice. Similarly, some weedy rice populations, such as Am-3, Po-2, Ku-3, and Pu-2 also shared a relatively high proportion of genetic cluster assignment with each other, suggesting the frequent gene flow between these weedy rice populations.

In addition, when genetic structure of each of the weedy rice populations was plotted to the geographical locations, the proportion of genetic components of different weedy rice populations showed a pattern of somewhat graduate change from south to north in Sri Lanka, i.e., red in northern populations and green in southern populations. This indicated south-to-north genetic differentiation of weedy rice populations. However, no such differentiation was obviously observed between western and eastern populations or between the Wet and Dry zones (Figure 1).

## Discussion

### Abundant within-population genetic diversity and limited population differentiation

Understanding genetic diversity and the possible mechanisms contributing to the formation and maintenance of such diversity within and among weedy rice populations is essential for designing strategies for the effective control of this weed [5],[11]. Results from our analyses showed a high level of genetic diversity in Sri Lankan weedy rice populations as estimated by 23 SSR loci, which is comparable with that estimated based on morphological characterization [9]. The overall within-population genetic diversity was unexpectedly high for such a self-pollination plant as weed rice, suggesting possibly a high level of gene flow, which is commonly observed in outbreeding species. Compared to previous studies with weedy rice populations from northeast China [11], USA [34], and northern Italy [6], genetic diversity in this study as estimated also by SSR fingerprints was much greater, even though the geographic coverage of the sampled weedy rice populations in other studies [11],[34] was much wider. It is widely recognized that abundant genetic diversity allows weeds, including weedy rice, to adapt to a broad range of environmental conditions, such as the change of habitat temperature, change of farming styles, and human disturbance [11],[35],[36], causing problems for agriculture production. The abundant genetic diversity of weedy rice in Sri Lanka may contribute to its rapid spread (along with the contaminated rice cultivars) and adaptation across various rice planting regions. The current situation of increased damages of rice yield by weedy rice as reported by Marambe & Amarasinghe [18] in Sri Lanka may also be explained by the increased genetic diversity of weedy rice. In addition, as reported by Dai *et al*., rich genetic diversity of weedy rice can significantly enhance its competition ability over cultivated rice [8]. Obviously, increased genetic diversity can promote adaptive evolution and the competitive ability of weedy plant populations.

AMOVA revealed much greater genetic diversity within the Sri Lankan weedy rice populations (83%) than that among populations (15%) and between the two zones (2%). These results suggest that genetic diversity of Sri Lankan weedy rice mainly exists within populations, with limited genetic differentiation among populations, which gains supports from the relatively low *F_st_* value. Previous studies from China, USA, and Italy [6],[11],[35] demonstrated a higher level of genetic differentiation among weedy rice populations. Such a pattern of limited genetic differentiation is unusual for Sri Lankan weedy rice, which is a self-pollination plant. The only explanation could be exceed gene flow among populations.

The diversity pattern of Sri Lankan weedy rice, having abundant within population genetic diversity and limited genetic differentiation, suggests considerable level of gene flow, which is an important driving force to homogenize differences among populations [37]. In other words, a considerable amount of gene flow conveying diverse genotypes of weedy rice from different sources has increased genetic diversity of weedy rice occurring in the same field, and reduced genetic differentiation of weedy rice populations among fields or regions. Considering the traditional practices in which farmers frequently exchange uncertified rice seeds between different fields and regions in Sri Lanka, we considered that the long-distance movement of weedy rice seeds mixed in cultivated rice seeds may have played an important role in increasing the within-population genetic diversity and reducing the among-population genetic differentiation.

### Limited spatial genetic structure of weedy rice populations caused by seed-mediated gene flow

Usually, the formation of a strong genetic structure of plant populations at a given spatial region is associated with limited gene flow [38], mediated either by pollination or seed dispersal. Knowledge on spatial genetic structures of populations can facilitate our understanding of the pathways of weedy rice gene flow. The presence of weedy rice can be greatly influenced by human activities associated with the style of rice farming [39], such as the sources and exchanging modes of rice seeds that may possibly be contaminated by weedy rice.

Our results from UPGMA cluster and Mantel test suggested little association between genetic relatedness and spatial location of weedy rice populations. In other words, weedy rice populations with close spatial locations did not show close genetic relationships, and *vice versa*. For example, the Ma-1 population from the Matara District was genetically more closely related to populations (Am-2 and Am-4) from another distant District than populations from its own District. In contrast, some spatially close populations (e.g., Ma-1, Ma-2, and Ma-4) showed a distant genetic relationship. All these results suggested a loose genetic structure of Sri Lankan weedy rice populations, most likely caused by substantial gene flow. Data from the analysis of pair-wise migration among the 21 weedy rice populations confirmed our hypothesis of a high level of gene flow, up to 122 migrants per generation. Even between populations (e.g., Ma-1 and Am-4) with a great spatial distance, the detected gene flow can be as high as ∼1.3 migrants per generation (see Table 5 for details). Given the strong seed productive ability of weedy rice [5], such a level of immigration will significantly promote the increase in weedy rice populations in a given rice field.

As a relatively strict self-pollination plant, weedy rice has extremely low frequency of pollen-mediated gene flow [12],[40] and a limited pollen travel distance (<10 m as referred by its conspecific cultivated rice) [41]. Therefore, we propose that the observed gene flow is most likely caused by the long-distance (kilometers) dispersal of cultivar seeds, in which weedy rice is mixed [5]. Given that the long-distance exchange of uncertified rice seeds among farmers is a common practice in Sri Lanka, it is greatly possible for weedy rice to be spread across the entire rice planting regions along with the long-distance travel of cultivated rice seeds. The exceed gene flow mediated by seed dispersal can well explain the phenomenon of the limited genetic differentiation among Sri Lankan weedy rice populations across the country. In fact, the determined average gene flow (migrants) level for a particular weedy rice population is significantly positively correlated with the level of its expected heterozygosity (*H_e_*), indicating the importance of gene flow to create and accumulate within-population genetic diversity in Sri Lankan weedy rice populations.

Results based on the STRUCTURE analysis also strongly support our hypothesis of exceed gene flow among different rice planting regions, which leads to the limited spatial genetic structure of Sri Lankan weedy rice populations. The profile of STRUCTURE analysis indicates the considerable sharing of genetic components among different rice planting regions. For example, the northern weedy rice populations, Po-2 (from Polonnaruwa), Pu-1 and Pu-2 (from Puttalam), and Am-4 (from Ampara), shared considerable amount of genetic components with the southern populations from Matara District, obviously suggesting the exchange of genetic materials among populations across these districts. STRUCTURE analysis did not suggest an obvious admixture of different genetic components in weedy rice populations. These results, together with the extremely low observed heterozygosity and high fixation index, clearly indicated the lack of hybridization, which is usually caused by pollen-mediated gene flow, between weedy rice populations, although the possibility of crop-to-weed pollen-mediated gene flow within the same rice fields cannot be excluded [12],[40]. The profile of genetic structure for weedy rice populations obtained from this study adequately reflects the current practices in Sri Lanka of the long-distance distribution of uncertified rice seeds in which weedy-rice seeds are contaminated. All together, these results strongly support our hypothesis of seed-mediated gene flow that has caused the increased genetic diversity within weedy rice populations, as also reported in other studies [39],[42], and reduced genetic differentiation among weedy rice populations, as happened in cultivated rice [39], which has resulted in their limited spatial genetic structure. Apparently, seed-mediated gene flow is an important reason that leads to the rapid dispersal and infestation of weedy rice in Sri Lanka.

### Implications for weedy rice control and management

To ensure the sustainable rice production, effective weedy rice control and management is critical in rice ecosystems where the infestation of weedy rice becomes inevitable. Weedy rice seeds can be accidentally harvested alone with the harvested cultivar seeds and spread to other rice fields through cultivar-seed distribution when these seeds are used for planting in the next seasons [5]. Understanding the patterns of genetic diversity and structure of weedy rice populations will help us to predict their potential gene flow in a given region, in addition to designing useful control measures for weedy rice in different geographical locations. There are many existing control measures, such as crop rotation, transplanting of cultivated rice, mechanical weed control, and proper chemical (e.g., herbicide) application according to diverse types of weedy rice that infest rice fields [5]. Managing the distribution of cultivated rice seeds that are contaminated by weedy rice can prevent introducing weedy rice seeds into clean paddy fields or less infested fields achieved by applying other effective weed control measures. Once being introduced into a paddy field from other regions through seed contamination, weedy rice can quickly establish its populations in a few generations through seed reproduction and accumulation in soil seed-banks [5], making the eradication of this weed very difficult.

Our findings of extensive seed-mediated gene flow in this study not only provide reasonable explanations for the increased genetic diversity within populations and reduced genetic differentiation among populations in Sri Lankan weedy rice, but also have important implications for weedy rice control in this country (also in other countries having the similar rice farming styles). As indicated, it is common that Sri Lankan rice farmers save their own rice seeds for planting in the next generation. The exchange of the saved rice seeds between different villages and districts is the common practice for seed distribution in Sri Lanka. Given the situation of weedy rice infestation in Sri Lanka and our findings from this study, we consider that the current rice seed distribution pattern is largely responsible for the rapid spread of weedy rice across the entire country, causing serious infestation. Therefore, we propose to break the long-distance movement and spread of weedy rice by only using certified clean rice seeds provided either by the government agencies or licensed seed production companies. This measure will be a key step in the weedy rice control program in combination with other effective methods. Without this step, the continual introduction of weedy rice through contaminated rice seeds will make the control of this weed very difficult, even though other measures are effective. Therefore, clean seed sources without the contamination of weedy rice can significantly minimize the spread and infestation of this weed.

Currently in Sri Lanka, 90% of rice seed supplies are provided by farmers' self-saved seeds, and the distribution of rice seeds is essentially on a farmer-to-farmer basis. In the entire country, only ∼10% of the certified seeds are provided and distributed by government-based seed production agencies, e.g. Department of Agriculture (4%), and private seed production companies (6%) [43]. The Sri Lankan government has attempted to increase the proportion of certified rice seed uses to ∼20%. However, due to various reasons, this goal has not been reached so far. We strongly recommend that the use and distribution of certified clean rice seeds should gradually replace the current model of self-saved seed use and farmer-to-farmer seed distribution, to significantly minimize the rice yield damage caused by the infestation of weedy rice. Actually, the use of certified rice seeds should be more economic in rice production for farmers in a long run, because of the potential threats of rice yield losses (up to 90%) [18] from weedy rice infestation. Probably, it sounds more costly for farmers to grow rice by purchasing certified seeds than using the saved seeds that are essentially free, but the substantial yield losses caused by weedy rice infestation through mixed weed in seed sources will result much higher prices for farmers. In addition, fields used for producing certified rice seeds that are distributed to farmers for cultivation should be closely monitored to ensure no weedy rice infestation. Integrated weed control measures including those recommended above can ensure the safe and sustainable production of rice in Sri Lanka significantly, by minimizing rice yield damages from weedy rice infestation.

## Supporting Information

Table S1 **Pair-wise genetic differentiation among the collected weedy rice populations and cultivated rice.** The genetic differentiation was estimated by *F_st_* values calculated based on the FSTAT software [25].
(DOCX)

Table S2 **Pair-wise standardized genetic differentiation among the collected weedy rice populations and cultivated rice.** The genetic differentiation was estimated by the *F′_st_* values obtained based on the division of the original *F_st_* values by the *F_st_*-estimator (maximum value) [25],[26].
(DOCX)
