# Supplementary material for: Seed-Mediated Gene Flow Promotes Genetic Diversity of Weedy Rice within Populations: Implications for Weed Management
Source: PLoS One. 2014 Dec 1;9(12):e112778. doi: 10.1371/journal.pone.0112778 (PMC4249867; doi:10.1371/journal.pone.0112778)
Supplement: Table S1 — Pair-wise genetic differentiation among the collected weedy rice populations and cultivated rice. The genetic differentiation was estimated by Fst values calculated based on the FSTAT software [25]. (DOCX) [file pone.0112778.s001.docx]

Table S1.

|  | Am-1 | Am-2 | Am-3 | Am-4 | Po-1 | Po-2 | An-1 | An-2 | An-3 | An-4 | An-5 | An-6 | Ku-1 | Ku-2 | Ku-3 | Ma-1 | Ma-2 | Ma-3 | Ma-4 | Pu-1 | Pu-2 | CV |
| --- | --- | --- | --- | --- | --- | --- | --- | --- | --- | --- | --- | --- | --- | --- | --- | --- | --- | --- | --- | --- | --- | --- |
| Am-1 | - |  |  |  |  |  |  |  |  |  |  |  |  |  |  |  |  |  |  |  |  |  |
| Am-2 | 0.11 | - |  |  |  |  |  |  |  |  |  |  |  |  |  |  |  |  |  |  |  |  |
| Am-3 | 0.11 | 0.07 | - |  |  |  |  |  |  |  |  |  |  |  |  |  |  |  |  |  |  |  |
| Am-4 | 0.09 | 0.06 | 0.08 | - |  |  |  |  |  |  |  |  |  |  |  |  |  |  |  |  |  |  |
| Po-1 | 0.14 | 0.13 | 0.13 | 0.14 | - |  |  |  |  |  |  |  |  |  |  |  |  |  |  |  |  |  |
| Po-2 | 0.10 | 0.06 | 0.07 | 0.06 | 0.08 | - |  |  |  |  |  |  |  |  |  |  |  |  |  |  |  |  |
| An-1 | 0.33 | 0.28 | 0.29 | 0.31 | 0.23 | 0.22 | - |  |  |  |  |  |  |  |  |  |  |  |  |  |  |  |
| An-2 | 0.26 | 0.23 | 0.22 | 0.25 | 0.17 | 0.16 | 0.002 | - |  |  |  |  |  |  |  |  |  |  |  |  |  |  |
| An-3 | 0.15 | 0.17 | 0.17 | 0.15 | 0.09 | 0.10 | 0.27 | 0.18 | - |  |  |  |  |  |  |  |  |  |  |  |  |  |
| An-4 | 0.10 | 0.06 | 0.09 | 0.12 | 0.05 | 0.04 | 0.19 | 0.14 | 0.09 | - |  |  |  |  |  |  |  |  |  |  |  |  |
| An-5 | 0.21 | 0.23 | 0.19 | 0.23 | 0.10 | 0.15 | 0.31 | 0.26 | 0.23 | 0.17 | - |  |  |  |  |  |  |  |  |  |  |  |
| An-6 | 0.21 | 0.18 | 0.16 | 0.17 | 0.12 | 0.07 | 0.24 | 0.19 | 0.22 | 0.13 | 0.16 | - |  |  |  |  |  |  |  |  |  |  |
| Ku-1 | 0.18 | 0.17 | 0.18 | 0.16 | 0.07 | 0.09 | 0.26 | 0.20 | 0.15 | 0.09 | 0.17 | 0.12 | - |  |  |  |  |  |  |  |  |  |
| Ku-2 | 0.13 | 0.12 | 0.15 | 0.15 | 0.11 | 0.07 | 0.30 | 0.24 | 0.15 | 0.05 | 0.18 | 0.16 | 0.07 | - |  |  |  |  |  |  |  |  |
| Ku-3 | 0.13 | 0.09 | 0.12 | 0.10 | 0.05 | 0.04 | 0.12 | 0.08 | 0.06 | 0.02 | 0.15 | 0.10 | 0.06 | 0.08 | - |  |  |  |  |  |  |  |
| Ma-1 | 0.11 | 0.09 | 0.17 | 0.06 | 0.152 | 0.11 | 0.32 | 0.28 | 0.16 | 0.14 | 0.24 | 0.21 | 0.15 | 0.14 | 0.13 | - |  |  |  |  |  |  |
| Ma-2 | 0.14 | 0.13 | 0.18 | 0.13 | 0.14 | 0.11 | 0.33 | 0.26 | 0.12 | 0.12 | 0.24 | 0.23 | 0.14 | 0.11 | 0.12 | 0.11 | - |  |  |  |  |  |
| Ma-3 | 0.13 | 0.13 | 0.17 | 0.13 | 0.12 | 0.10 | 0.30 | 0.23 | 0.07 | 0.10 | 0.23 | 0.22 | 0.11 | 0.08 | 0.09 | 0.11 | 0.001 | - |  |  |  |  |
| Ma-4 | 0.23 | 0.24 | 0.19 | 0.21 | 0.15 | 0.09 | 0.31 | 0.24 | 0.21 | 0.16 | 0.23 | 0.06 | 0.13 | 0.20 | 0.12 | 0.25 | 0.25 | 0.24 | - |  |  |  |
| Pu-1 | 0.22 | 0.22 | 0.23 | 0.22 | 0.07 | 0.17 | 0.29 | 0.23 | 0.19 | 0.12 | 0.18 | 0.22 | 0.12 | 0.18 | 0.08 | 0.25 | 0.23 | 0.23 | 0.25 | - |  |  |
| Pu-2 | 0.13 | 0.08 | 0.11 | 0.10 | 0.05 | 0.04 | 0.19 | 0.13 | 0.09 | 0.03 | 0.16 | 0.10 | 0.07 | 0.11 | 0.004 | 0.14 | 0.15 | 0.14 | 0.09 | 0.10 | - |  |
| CV | 0.17 | 0.23 | 0.25 | 0.24 | 0.25 | 0.20 | 0.40 | 0.34 | 0.25 | 0.15 | 0.32 | 0.30 | 0.26 | 0.17 | 0.21 | 0.27 | 0.25 | 0.25 | 0.33 | 0.30 | 0.21 | - |
