# Supplementary material for: Seed-Mediated Gene Flow Promotes Genetic Diversity of Weedy Rice within Populations: Implications for Weed Management
Source: PLoS One. 2014 Dec 1;9(12):e112778. doi: 10.1371/journal.pone.0112778 (PMC4249867; doi:10.1371/journal.pone.0112778)
Supplement: Table S2 — Pair-wise standardized genetic differentiation among the collected weedy rice populations and cultivated rice. The genetic differentiation was estimated by the F′st values obtained based on the division of the original Fst values by the Fst-estimator (maximum value) [25],[26]. (DOCX) [file pone.0112778.s002.docx]

Table S2.

|  | Am-1 | Am-2 | Am-3 | Am-4 | Po-1 | Po-2 | An-1 | An-2 | An-3 | An-4 | An-5 | An-6 | Ku-1 | Ku-2 | Ku-3 | Ma-1 | Ma-2 | Ma-3 | Ma-4 | Pu-1 | Pu-2 | CV |
| --- | --- | --- | --- | --- | --- | --- | --- | --- | --- | --- | --- | --- | --- | --- | --- | --- | --- | --- | --- | --- | --- | --- |
| Am-1 | - |  |  |  |  |  |  |  |  |  |  |  |  |  |  |  |  |  |  |  |  |  |
| Am-2 | 0.26 | - |  |  |  |  |  |  |  |  |  |  |  |  |  |  |  |  |  |  |  |  |
| Am-3 | 0.26 | 0.16 | - |  |  |  |  |  |  |  |  |  |  |  |  |  |  |  |  |  |  |  |
| Am-4 | 0.20 | 0.12 | 0.17 | - |  |  |  |  |  |  |  |  |  |  |  |  |  |  |  |  |  |  |
| Po-1 | 0.35 | 0.29 | 0.28 | 0.31 | - |  |  |  |  |  |  |  |  |  |  |  |  |  |  |  |  |  |
| Po-2 | 0.26 | 0.13 | 0.15 | 0.15 | 0.19 | - |  |  |  |  |  |  |  |  |  |  |  |  |  |  |  |  |
| An-1 | 0.64 | 0.50 | 0.52 | 0.57 | 0.43 | 0.43 | - |  |  |  |  |  |  |  |  |  |  |  |  |  |  |  |
| An-2 | 0.57 | 0.46 | 0.43 | 0.50 | 0.34 | 0.34 | -0.004 | - |  |  |  |  |  |  |  |  |  |  |  |  |  |  |
| An-3 | 0.36 | 0.38 | 0.37 | 0.33 | 0.21 | 0.22 | 0.48 | 0.35 | - |  |  |  |  |  |  |  |  |  |  |  |  |  |
| An-4 | 0.29 | 0.16 | 0.24 | 0.30 | 0.15 | 0.11 | 0.39 | 0.33 | 0.26 | - |  |  |  |  |  |  |  |  |  |  |  |  |
| An-5 | 0.45 | 0.45 | 0.36 | 0.45 | 0.19 | 0.32 | 0.52 | 0.46 | 0.45 | 0.39 | - |  |  |  |  |  |  |  |  |  |  |  |
| An-6 | 0.47 | 0.37 | 0.31 | 0.36 | 0.25 | 0.15 | 0.42 | 0.36 | 0.47 | 0.31 | 0.30 | - |  |  |  |  |  |  |  |  |  |  |
| Ku-1 | 0.41 | 0.36 | 0.37 | 0.33 | 0.15 | 0.20 | 0.46 | 0.39 | 0.32 | 0.22 | 0.32 | 0.23 | - |  |  |  |  |  |  |  |  |  |
| Ku-2 | 0.30 | 0.27 | 0.33 | 0.32 | 0.25 | 0.17 | 0.55 | 0.49 | 0.34 | 0.13 | 0.36 | 0.34 | 0.14 | - |  |  |  |  |  |  |  |  |
| Ku-3 | 0.34 | 0.22 | 0.29 | 0.25 | 0.13 | 0.12 | 0.24 | 0.17 | 0.16 | 0.07 | 0.34 | 0.24 | 0.14 | 0.21 | - |  |  |  |  |  |  |  |
| Ma-1 | 0.25 | 0.19 | 0.33 | 0.12 | 0.31 | 0.23 | 0.56 | 0.52 | 0.34 | 0.33 | 0.45 | 0.40 | 0.29 | 0.29 | 0.29 | - |  |  |  |  |  |  |
| Ma-2 | 0.32 | 0.28 | 0.38 | 0.27 | 0.29 | 0.25 | 0.59 | 0.51 | 0.25 | 0.30 | 0.46 | 0.45 | 0.29 | 0.23 | 0.28 | 0.21 | - |  |  |  |  |  |
| Ma-3 | 0.31 | 0.28 | 0.35 | 0.28 | 0.26 | 0.22 | 0.51 | 0.43 | 0.15 | 0.26 | 0.44 | 0.44 | 0.23 | 0.18 | 0.22 | 0.22 | 0.002 | - |  |  |  |  |
| Ma-4 | 0.48 | 0.46 | 0.36 | 0.40 | 0.29 | 0.19 | 0.52 | 0.42 | 0.41 | 0.37 | 0.40 | 0.11 | 0.25 | 0.40 | 0.27 | 0.45 | 0.47 | 0.44 | - |  |  |  |
| Pu-1 | 0.49 | 0.46 | 0.47 | 0.44 | 0.14 | 0.39 | 0.50 | 0.44 | 0.40 | 0.31 | 0.33 | 0.45 | 0.25 | 0.40 | 0.18 | 0.48 | 0.46 | 0.47 | 0.47 | - |  |  |
| Pu-2 | 0.34 | 0.18 | 0.26 | 0.23 | 0.13 | 0.09 | 0.36 | 0.28 | 0.23 | 0.09 | 0.35 | 0.23 | 0.16 | 0.27 | -0.01 | 0.31 | 0.35 | 0.32 | 0.19 | 0.22 | - |  |
| CV | 0.45 | 0.56 | 0.60 | 0.59 | 0.63 | 0.53 | 0.79 | 0.77 | 0.64 | 0.45 | 0.69 | 0.68 | 0.62 | 0.43 | 0.59 | 0.61 | 0.59 | 0.60 | 0.70 | 0.70 | 0.58 | - |
